# *Schistosoma mansoni* and soil-transmitted helminths among preschool-aged children in Chuahit, Dembia district, Northwest Ethiopia: prevalence, intensity of infection and associated risk factors

**DOI:** 10.1186/s12889-016-2864-9

**Published:** 2016-05-23

**Authors:** Agersew Alemu, Yalewayker Tegegne, Demekech Damte, Mulugeta Melku

**Affiliations:** Department of Medical Parasitology, School of Biomedical and Laboratory Sciences, College of Medicine and Health Sciences, University of Gondar, Gondar, Ethiopia; Gondar University Hospital, College of Medicine and Health Sciences, University of Gondar, Gondar, Ethiopia; Department of Hematology and Immunohematology, School of Biomedical and Laboratory Sciences, College of Medicine and Health Sciences, University of Gondar, Gondar, Ethiopia

**Keywords:** Associated risk factor, Ethiopia, Intensity of infection, Preschool-aged children, *Schistosoma mansoni*, Soil-transmitted helminthes

## Abstract

**Background:**

Intestinal schistosomiasis and soil-transmitted helminthiasis are the major public health problems globally. Compared with any other age group, pre-school aged children and school-aged children are the most exposed. There are few studies showing the burden of intestinal schistosomiasis, and soil-transmitted helminthiasis among pre-school aged children in Ethiopia. Hence, this study aimed to assess the prevalence of *schistosoma mansoni* and soil-transmitted helminths and associated risk factors among preschool aged children of Chuahit and surrounding Kebeles, Northwest Ethiopia.

**Methods:**

A community based cross sectional study was conducted from February 2 to March 27 2015. Four hundred one preschool-aged children were included in the study by using two stage cluster sampling technique. Pretested structured questionnaire was employed to collected data via face-to-face interview technique. A single stool specimen was collected, and a portion of the sample was processed by Kato Katz method.

**Results:**

Of the total children, 141 (35.2 %) harbored one or more intestinal helminthes. *Schistosoma mansoni* was found in 45 (11.2 %) of preschool age children. *Ascaris lumbricoides* was the predominant isolate, 77 (19.2 %) followed by *S. mansoni,* 45 (11.2 %). The least parasites isolated were *Tania species,* 2 (0.5 %). After adjusting for other variables, being mothers who did not have the habit of washing hands after toilet (AOR = 7.3, 95%CI: 2.97–17.95), being occupationally housewife mothers (AOR = 8.9, 95%CI: 2.27–25.4), using protected spring water as a main family source of water (AOR = 3.9, 95%CI: 1.2–12.3) and child habit of not wearing shoe (AOR = 1.91, 95%CI: 1.01–3.64) were significantly associated with high prevalence of soil-transmitted helminthiasis among preschool-age children in Chuahit.

**Conclusion:**

The current study showed that relatively higher level of STH and *S. mansoni* among preschool-aged children in Chuahit. This finding calls for a need of public health education, promotion of women education and provision of safe water to reduce the burden of soil-transmitted intestinal helminthiasis and schistosomiaisis.

## Background

Intestinal schistosomiasis and soil-transmitted helminthiasis are the major medical and public health problems in many parts of the world. Schistosomiasis remains a serious public health concern in sub-Saharan Africa (SSA) and approximately one-third of the 192 million cases of schistosomiasis in the SSA are caused by Schistosoma mansoni, the causal agent of intestinal schistosomiasis [1].

Soil-transmitted helminths (STH) of major concern to humans are *Ascaris lumbricoides, Trichuris trichiura, Necator americanus* and *Ancylostoma duodenale*. Infection results from ingestion of eggs and contact with fecally contaminated soil, and occurs primarily in areas where the sanitation is poor sanitation and water supplies are unsafe. The latest estimates indicate that more than 2 billion people are infected with these parasites [2].

Schistosomiasis is a chronic Neglected Tropical parasitic disease (NTD) caused by schistosome species (*S. haematobium, S. mansoni, S. japonicum, S. mekongi and S. intercalatum*) found in fresh water, and human beings that come into contact with fresh water containing fresh water snails are at risk for infection. Two hundred forty million people are already infected with schistosomiasis and 700 million are at risk in 74 countries. Schistosomiasis ranks the second most common parasitic disease, and it is the fatal NTD killing an estimated 280,000 people each year in the African region alone in which more than 90 % of the cases occur [3]. Recognizing the public health impact of schistosomiasis and soil-transmitted helminthiasis, the World Health Organization (WHO) has set a minimum target for the control of morbidity due to these parasitic worm infections, urging member states to regularly treat at least 75 % of all school-aged children (SAC) at risk of morbidity [4].

Control of STH and intestinal Schistosomiasis at population intervention level primarily bases on regular anti-helminthic and praziquantel treatment, health education, improving sanitation standards and developing a control strategy. These population based intervention are better than individual treatment and environmental measures like snail control in areas where Schistosomiasis common [5]. In developing countries, however, control measures are difficult to implement due to inadequate clear water supply, poor sanitation and limited access to education. As a result, intestinal helminthic infection remains a significant health problem in these regions [6].

Ethiopia has one of the lowest quality-drinking water supply and latrine coverage in the world [6, 7]. As a result, Schistosomiasis and STH are endemic in many parts of Ethiopia, and the intestinal form of schisosomiasis caused by *S. mansoni* is more widely distributed in the country compared to the urinary form caused by *S. hamatobium* which is limited to some lowland areas of Ethiopia [8].

Morbidity caused by *S. mansoni* and STH is commonly associated with heavy infection intensities. Compared with any other age group, preschool-aged children (PSAC) and (SAC) are the most exposed groups, and they harbor the greatest numbers of intestinal worms resulting in diarrhea, loss of appetite, weight loss, growth retardation, malnutrition, anemia, cognitive defects and hepatosplenomegaly in chronic cases which may lead to sever heath problem and mortality later in life [9–11].

Schistosomiasis in African PSAC has been previously unnoticed but much better recognized in recent years. Although increasing evidence showing that treatment with praziquantel is safe, beneficial, and could be delivered within ongoing public health interventions, PSAC do not have satisfactory access to this drug, and there is a treatment gap [12, 13]. Most mass drug administration (MDA) campaigns have been implemented in sub-Saharan Africa treating millions of SAC for schistosomiasis with praziquantel (PZQ). But PSAC (≤7 years) have been every time excluded from access to such kind of medication [14]. The reason behind is the belief that PSAC would not yet been exposed to infested freshwater bodies, thus the extent and severity of schistosomiasis is not sufficiently understood and documented in this age class [15]. Recent studies showed that the prevalence of schistosome infections is high among PSAC whose parents have high levels of contact with water [13, 16]. Studies which were conducted in Mali, Niger, Sudan, Uganda and Zimbabwe showed that PSAC are at high risk of schistosomiasis, and prevalence of the infection ranged from 18 to 63 % which implies that PSAC were at high-risk in areas where schistosomiasis is endemic [17]

In Ethiopia, intestinal parasitic infections are highly prevalent: 5.01 million are infected with schistosomiasis and 37.5 million to be at risk. The burden and intensity of infection vary from locality to locality.. The estimated number of peoples infected with *hookworm*, *A.lumbricoides* and *T.trichiura* respectively were 11 million, 26 million and 21 million respectively [18]. A study done in Wondo Genet, Ethiopia showed that the prevalence of *T.trichiura*, *S.mansoni*, *A.lumbricoides*, *Hymenolepis nana*, and hookworm infections were 74.7, 37.2, 25.7, 4.5, and 5.9 %, respectively [19]. In another similar study done in Wonji Shoa, Ethiopia, the prevalence of *T.trichiura*, *S.mansoni*, *A. lumbricoides*, *Hymenolepis nana*, and hookworm infections were 2.9, 8.8, 4.6, 10.4, 0.8 % respectively [20].

Most previous studies conducted in Ethiopia were focusing on school-age children and only few studies reported intestinal helminths infections among preschool-aged children. Thus, the current study was undertaken to assess the prevalence of *S.mansoni*, STH infections and associated risk factors among preschool-aged children in Denbia district, North West Ethiopia.

## Methods

### Study design, area and population

A Community based cross-sectional study was conducted in preschool aged children living in Chuahit, Dembia District, North West Ethiopia, from February 2 to March 27, 2015. Chuahit is found in North Gondar Zone of Amhara Region of Ethiopia and is located 789 kms Northwest of the capital city Addis Ababa. It is one of the four small rural town found in Dembia District. Under its administrative area, it comprises nine kebeles. Four rivers namely Tanti Kura, Ambizina, Ambagenin and Chigero flow through it. The average temperature and humidity are 28 °C and 22 % respectively. PSAC who were under the age of seven years old were the source population [17]. PSAC who had been living in Chuahit for at least 6 months before the study began and who had no history of having been treated with Praziquantel (PZQ) and anti-helminthic drug in the past 4 weeks were included.

### Sample size determination and sampling technique

The required sample size was determined by using single population proportion formula with the following assumptions: prevalence (p) of 15.5 % from a previous study [20], 95 % confidence level, 5 % margin of error, design effect of 2. Accordingly, the minimum sample size (n) was found to be 401. A multi-stage sampling procedure was utilized to select study participants. At the first stage, two kebeles (Derena and Meskele kerestos) were selected randomly by simple random sampling from nine kebeles. At the second sampling stage, numbers of households included in each kebeles were determined by proportional allocation according to the total number of households found in each kebeles. Then, systematic sampling method was employed to select the households. The sampling interval was calculated by dividing the total households to the number of households to be included in the sample for each kebeles. The initial household interviewed was randomly selected by lottery method and the next households were selected at that interval. Whenever more than one eligible children were found in the same selected household, only one of them was chosen using the lottery method. In case no eligible candidate was identified in a selected household, the next household was selected keeping the interval constant afterwards. According to Chuahit administrative health office report, 6,947 PSAC were estimated to reside in Chuahit; of whom 781 and 523 were residing in Derena and Meskele kerestos respectively. Furthermore, 2,820 households (1462 in Derena and 1358 in Meskele kerestos) were found in the selected kebeles. Two hundred eight and 193 households were selected from the Derena and Meskele kerestos kebeles respectively. Likewise, 208 and 193 PSAC were participated in the study from Derena and Meskele kerestos kebeles respectively.

### Data collection and processing

#### Questionnaire survey

Data were collected by pretested structured Amharic version questionnaire using interview technique. Health extension workers were recruited for data collection and supervised by the Principal Investigator. Before data collection, data collectors were assigned to each Kebeles. Then each data collector conducted a house-to house survey. During the survey, the data collectors first asked the parents of PSAC whether or not any PSAC present or not in the household. When PSAC was found, the data collectors interviewed parents or caretakers after obtaining written informed consent. Repeat visits were conducted for those individuals who were not available during the first visit. At each data collection spot, full explanation about the aim of the research was given to the parents or caretakers of PSAC before the interview.

### Sample collection and laboratory procedures

A single stool specimen of about 5 g was collected from each study participant using clean, dry and leak proof plastic container labeled with unique identification number. A portion of the sample was processed by Kato Katz method using a template holding 41.7 mg of stool [21]. Examination for hookworm was performed within one hour of stool collection and Kato slide preparation. The slides were left for 24 h to clear for easy visualization of *S. mansoni* and other helminthes eggs. After 24 h, two experienced laboratory technologists examined each slides independently. Whenever the result of the two laboratory technologists discorded, the third experienced laboratory technologist examined the slides. Results of the laboratory investigation were recorded on a format prepared for this purpose. Infection intensity of the STHs and *S. mansoni* was estimated by multiplying the total number of eggs counted by 24, which gives as the eggs per gram (epg) of stool. Besides, the species specific classes of infection intensity with *S.mansoni* and STH were classified as light, moderate and heavy per the threshold set by WHO [22].

### Data management and analysis

Data were entered in to and analyzed by statistical package for social sciences (SPSS) statistical software version 20. Study findings were explained in words, tables and other statistical summary techniques. Binary logistic regression model was used to identify factors associated with infection of *S. mansoni* and STHs. Multiple logistic regressions was fitted to control the possible effect of confounders, and finally the variables having independent association with dependent variables were identified on the basis of odd ratio (OR) with 95 % confidence interval (CI) and *P-value* less than 0.05.

### Ethical considerations

The study was conducted after ethical approval was obtained from institutional ethical review committee of College of Medicine and Health Sciences, University of Gondar. Moreover, letter of support was secured from the Woreda health office and each Kebele administrations. Following an explanation of the purpose, the benefits and the possible risks of the study, written consent was obtained from parents/care takers of PSAC, which assured that the participation was on voluntarily basis. Children who were positive for *S. mansoni* and STHs were treated by referring them to Chuahit health center.

## Results

### Socio-demographic characteristics of study participants

A total of 401 PSAC aged 6 months up to 6 years were included in the study. Out of these, 183 (45.6 %) were males and 218 (54.4 %) were females. The mean age of PSAC was 3.73 year (±1.75 year standard deviation). The majority of parents of PSAC were married, 374 (93.3 %); illiterate, 358 (89 %) and farmer, 388 (96.8 %) (Table 1).

**Table 1:** Socio-demographic characteristics of PSAC in Chuahit, Northwest Ethiopia, 2015

Variables	Character	Frequency	Percentage
Sex	Male	183	45.6
	Female	218	54.4
Age (in months)	≤12	49	12
	13–24	64	16
	25–36	83	20.7
	37–48	47	11.7
	49–60	67	16.7
	61–72	91	22.7
Marital status of mothers	Married	374	93.3
	Others	27	6.7
Educational status of mothers	Illiterate	358	89
	Read and write	16	4
	Primary school	27	6.7
Occupation of mothers	Farmer	388	96.8
	House wife	13	3.2

### Prevalence of *S. mansoni* and STHs

Of the total PSAC examined using single Kato-Katz method, 141 (35.2 %) had been infected with intestinal helminthes; 123 (87.2 %) with single and 18 (12.8 %) with double intestinal helminthes. *S. mansoni* was isolated in 45 (11.2 %) of PSAC. Regarding STHs, *A. lumbricoides* was the predominant parasite, 77 (19.2 %) followed by hookworms, 9 (2.2 %) and *T. trichiura,* 7 (1.7 %). The least parasites isolated were *Tania species,* 2 (0.5 %). The prevalence of *S. mansoni* and *T. trichiura* had shown increment with age whereas the prevalence of A*. lumbricoides* is highly prevalent in the age group 13–24 months. Prevalence of one or more intestinal helminthic infections showed significant association with age (p. value < 0.05). The prevalence of intestinal helminthic infection had not shown statistically significant difference between males and females. The overall prevalence of single and double parasitic infection was 123 (30.7 %) and 18 (4.5 %) respectively (Table 2). No PSAC is co-infected with three or more parasites. Co-infection rate was higher for *S. mansoni* and *A. lumbricoides* 5 (1.2 %) followed by *S. mansoni* and *H. nana* 4 (1 %).

**Table 2:** Prevalence of intestinal helminthiasis among PSAC in Chuahit, Northwest Ethiopia, 2015

Age(in months	Frequency	*S. mansoni*	*Hookworm*	*T.trichuris*	*A.lumbriciodes*	*E.vermicularis*	*H. nana*	*Taenia species*	One or more parasitic infection	Single parasitic infection	Double parasitic infection
≤12	49(12.2 %)	2(4.4 %)	0	0	4(5.2 %)	0	2(11.8 %)	0	8(5.7 %)	8(6.5 %)	0
13–24	64(16 %)	6(13.4 %)	1(11.1 %)	1(14.3 %)	16(20.8 %)	0	3(17.6 %)	0	24(17 %)	23(18.7 %)	2(11.1 %)
24- 36	83(20.7 %)	10(22.2 %)	3(33.4 %)	1(14.3 %)	18(23.4 %)	0	5(29.4 %)	1(50 %)	33(23.4 %)	27(22 %)	5(27.8 %)
37–48	47(11.7 %)	3(6.7 %)	1(11.1 %)	0	8(10.4 %)	0	2(11.8 %)	0	14(9.9 %)	14(11.4 %)	0
49–60	67(16.7 %)	11(24.4 %)	2(22.2 %)	2(28.6 %)	14(18.2 %)	2(66.7 %)	3(17.6 %)	1(50 %)	31(22 %)	27(22 %)	4(22.2 %)
61–72	91(22.7 %)	13(28.9 %)	2(22.2 %)	3(42.8 %)	17(22 %)	1(33.3 %)	2(11.8 %)	0	31(22 %)	24(19.5 %)	7(38.9 %)
Total	401(100 %)	45(11.2 %)	9(2.2 %)	7(1.7 %)	77(19.2 %)	3(0.7 %)	17(4.2 %)	2(0.5 %)	141(35.2 %)	123(30.7 %	18(4.5 %)
*x* ^2^(p value)		6.56(0.26)	2.14(0.83)	3.74(0.59)	5.85(0.32)	6.5(0.26)	1.63(0.9)	3.43(0.64)	12.82(**0.025**)	9.62(0.087)	7.78(0.17)
Sex											
Female	218(54.4 %)	22(48.9 %)	3(33.3 %)	6(85.7 %)	41(53.2.%)	1(33.3 %)	8(47.1 %)	1(50 %)	74(52.5 %)	66(53.7 %)	8(44.4 %)
Male	183(45.6 %)	23(51.1 %)	6(67.7 %)	1(14.3 %)	36(46.8 %)	2(67.7 %)	9(52.9 %)	1(50 %)	67(47.5 %)	57(46.3 %)	10(55.6 %)
Total	401(100 %)	45(11.2 %)	9(2.2 %)	7(1.7 %)	77(19.2 %)	3(0.7 %)	17(4.2 %)	2(0.5 %)	141(35.2 %)	123(30.7 %	18(4.5 %)
*x* ^2^(p value)		0.048(0.43)	1.64(0.2)	2.82(0.09)	0.61(0.43)	0.54(0.46)	0.38(0.54)	0.15(0.9)	0.31(0.58)	0.09(0.76)	0.75(0.39)

Bold p value indicates significant Pearson-chi square test at *P* < 0.05

### Infection intensity of *S. mansoni* and STHs

In this study, the mean egg per gram of faeces for individuals with detectable eggs of S*. manosni*, *A. lumbricoides*, *hookworm* and *T. trichiura* infections were 85 (Range: 24–288), 11,859 (Range: 96–118, 800), 61 (Range: 24–144) and 375 (Range: 24–2496) respectively. Among 45 PSAC who were positive for *S. mansoni,* 32 (71.1 %) and 13 (28.9 %) had light and moderate infection respectively. Among 77 PSAC who were positive for *A. lumbricoides*, 40 (51.9 %), 31 (40.3 %, 6 (7.8 %) had light, moderate and heavy intensity of infections. Moreover, except one PSAC who wass moderately infected with *T. trichiura,* the intensity of infection with *H.nana*, *hookworm, T. trichiura, E.vermicularis* and Taenia species was light (Table 3).

**Table 3:** Intensity of infection in PSAC with *S.mansoni* and soil-transmitted helminthic infection in Chuahit, Northwest Ethiopia, 2015

Intestinal Parasite	Class of infection intensity			
	light	Moderate	Heavy	total
*S.mansoni*	32(71.1 %)	13(28.9 %)	0	45(100 %)
*A.lumbricoides*	40(51.9 %)	31(40.3 %)	6(7.8 %)	77(100 %)
*H.nana*	17(100 %)	0	0	17(100 %)
*Hookworm*	9(100 %)	0	0	9(100 %)
*T.trichura*	6(85.7 %)	1(14.3 %)	0	7(100 %)
*E.vermicularis*	3(100 %)	0	0	3(100 %)
*Tania species*	2(100 %)	0	0	2(100 %)

### Risk factor analysis for soil-transmitted helminthiasis

In the present study, multivariate logistic regression analysis showed that the prevalence of soil-transmitted helminthiasis was not significantly associated with age of children, sex of children, maternal marital status and maternal educational status. However, prevalence of soil-transmitted helminthiasis was significantly associated with maternal habit of not washing hands after toilet (AOR = 7.3, 95 % CI: 2.97–17.95), occupationally housewife mothers (AOR = 8.9, 95 % CI: 2.27–25.4), the use of protected spring water as a main family source of water (AOR = 3.9, 95 % CI: 1.2–12.3) and children habit of not wearing shoe (AOR = 1.91, 95 % CI: 1.01–3.64) (Table 4).

**Table 4:** Bivariate ad multivariate logistic regression analysis of soil-transmitted helminthiasis in relation to selected variables among PSAC in Chuahit, Northwest Ethiopia, 2015

Variable	Total	Presence of soil-transmitted helminthiasis		COR(95 % CI)	AOR(95 % CI)
		Yes (%)	No (%)		
Child sex					
Male	183(45.6 %)	43(23.5 %)	140(76.5 %)	1.059(0.664–1.69)	
Female	218(54.4 %)	49(22.5 %)	169(77.5 %)	1	
Age(in months)					
≤ 12	49(12.2 %)	4(8.2 %)	45(91.8 %)	0.29(0.95–0.92)	
13–24	64(16 %)	18(28.1 %)	46(71.9 %)	1.3(0.62–2.7)	
25–36	83(20.7 %)	22(26.5 %)	61(73.5 %)	1.2(0.6–2.4)	
37–48	47(11.7 %)	9(19.1 %)	38(80.9 %)	0.79(0.33–1.99)	
49–60	67(16.7 %)	18(26.9 %)	49(73.1 %)	1.2(0.59–2.54)	
61–72	91(22.7 %)	21(23.1 %)	70(76.9 %)	1	
Marital status of mothers					
Married	374(93.3 %)	86(23)	288(77 %)	1.04(0.41–2.67)	
Divorced	27(6.7 %)	6(22.2 %)	21(77.8 %)	1	
Educational status of mothers					
Illiterate	358(89.3 %)	83(23.2 %)	275(76.8 %)	0.86(0.35–2.11)	
Read and write	16(4 %)	2(12.5 %)	14(87.5 %)	0.4(0.07–2.26)	
Primary school	27(6.7 %)	11(40.7 %)	16(59.3 %)	1	
Occupation of mothers					
Farmer	388(96.8 %)	85(21.9 %)	303(78.1 %)	1	1
House wife	13(3.2 %)	7(53.8 %)	6(46.2 %)	4.2(1.4–12.7)	8.9(2.27–34)*
Shoe wearing habit of the child					
Always	281(70.1 %)	50(17.8 %	231(82.2 %)	1	1
Sometimes	61(15.2 %)	21(34.4 %)	40(65.6 %)	2.43(1.32–4.47)	1.89(0.99–3.64)
Not at all	59(14.7 %)	21(35.6 %)	38(64.4 %)	2.6(1.38–4.72)	1.91(1.01–3.64)*
Source of water					
Protected spring	15(3.7 %)	8(53.3 %)	7(46.7 %)	4.2(1.45–11.65)	3.9(1.2–12.3)*
Well	386(96.3 %)	122(31.6 %)	264(68.4 %)	1	1
Hand washing habit after toilet					
Present	102(25.4 %)	6(5.9 %)	96(94.1 %)	1	1
Absent	299(74.6 %)	86(28.8 %)	213(71.2 %)	6.46(2.73–18.3)	7.34(2.9–15)*

*COR* crude odds ratio, *AOR* adjusted odds ratio, *CI* confidence interval, *statistically significant at *P* < 0.05 in multivariate logistic regression analysis

### Risk factor analysis for *S. mansoni*

In bivariate logistic regression analysis, habit of bringing the child to river, child washing habit in the river, child washing habit with freshly fetched water at home, child habit of swimming in river, high frequency swimming habit of child in the river per week, child habit of crossing the river with bare foot and low distance of home from the river were significantly associated with intestinal Schistosomiasis. However, in multivariate logistic regression analysis controlling the possible cofounders, none of them was significantly associated with intestinal Schistosomiasis in PSAC (Table 5).

**Table 5:** Bivariate and multivariate logistic regression analysis of intestinal *Schistosomiasis* in relation with selected variables among PSAC in Chuahit, Northwest Ethiopia, 2015

Variables	Presence of intestinal Schistosomiasis		Total (%)	COR(95 % CI)	AOR(85 % CI)
	Yes (%)	No (%)			
Sex of child					
Male	23(12.6)	160(87.4)	183(45.6)	1.28(0.69, 2.38)	
Female	2(10.1)	196(89.9)	218(54.4)	1	
Age of child (in Months)					
≤12	2(4.1)	47(95.9)	49(12.2)	1	
13–24	6(9.4)	58(90.6)	64(16)	2.43(0.47, 12.61)	
25–36	10(12)	73(88)	83(20.7)	3.22(0.68, 15.35)	
37–48	3(6.4)	44(94.6)	47(11.7)	1.60(0.26, 10.1)	
49–60	11(16.4)	56(83.6)	67(16.7)	4.62(0.97, 21.87)	
61–72	13(14.3)	78(85.7)	91(22.7)	3.92(0.85, 18.13)	
Maternal occupation					
Farmer	44(11.3)	344(88.7)	388(96.8)	1	
Housewife	1(7.7)	12(92.3)	13(3.2)	0.65(0.08, 5.13)	
Habit of bringing child to river					
Yes	43(21.1)	161(78.9)	204(50.9)	26(6.21, 109.15)*	2.86(0,16, 49.98)
No	2(1)	195(99)	197(49.1)	1	1
Habit of washing children in the river					
Yes	42(21.9)	150(78.1)	192(47.9)	19.23(5.84, 63.2)*	4.5(0.89, 4.1)
No	3(1.4)	20698.6)	209(52.1)	1	1
Habit of washing children with freshly fetched water at home					
Yes	42(21.1)	161(78.9)	204(50.9)	26.00(6.2, 109.14)*	6.70(0.98, 45.68)
No	2(1)	195(99)	197(49.1)	1	1
Habit of bringing children to irrigation sites					
Yes	1(14.3)	6(85.7)	7(1.7)	1.33(0.16, 11.27)	
No	44(11.2)	350(88.8)	394(98.3)	1	
Child’s swimming habit in river					
Yes	21(20.2)	83(79.8)	104(25.9)	2.88(1.52, 5.43)*	1.01(0.08, 12.3)
No	24(8.1)	273(91.9)	297(74.1)	1	
Child’s swimming frequency per week					
none	24(8.1)	273(91.9)	297(74.1)	1	1
1–2 times	9(18.4)	40(81.6)	49(12.2)	2.54(1.1, 5.85)*	1.01(0.33, 3.1)
≥3 times	12(21.8)	43(78.2)	55(14.2)	3.01(1.41, 6.45)*	1.15(0.38, 3.47)
Habit of child crossing river with bare foot					
Yes	25(18.9)	107(81.1)	132(32.9)	2.91(1.55, 5.45)*	0.6(0.29, 1.24)
No	20(7.4)	249(92.6)	269(67.10	1	1
Distance of home from the river					
<1 km	35(15.5)	191(84.5)	226(56.4)	3.02(1.45, 6.29)*	1.04(0.39, 2.77)
>1 km	10(5.7)	165(94.3)	175(43.6)	1	1

*Statistically significant at *P* < 0.05 in bivariate logistic regression analysis

### Mothers knowledge about *S. mansoni* and STH

Of the total 401 mothers (aged 20 up to 52 years) interviewed, 324 (80.8 %) and 166 (41.4 %) of (them had never heard about intestinal schistosomiasis and soil-transmitted helminthiasis respectively. For those mothers had ever heard of these parasitic infections, their major source of information was health professionals. From, mothers of PSAC interviewed about ways of transmission of intestinal schistosomiasis, 85 % of them responded that intestinal schistosomiasis cannot be transmitted by swimming or bathing in the river, crossing river with bare foot, washing clothes in river and fishing in river. Regarding preventive methods of intestinal schistosomiasis, 380 (94.8 %) of mothers did not know ways of prevention of intestinal schistosomiasis Regarding the symptoms of intestinal schistosomiasis, almost all the mothers, 377 (94 %) did not know the symptoms of the disease. Nearly half and two-third of the mothers responded that they did not know about ways of transmission and the preventive methods of STH respectively.

## Discussion

The prevalence of one or more intestinal helminths infections observed among the study participants was 141 (35.2 %). This finding is lower than study conducted in different area of Ethiopia namely; Methara (89 %) [23], Wondo Genet (85.1 %) [19], Tigray (48.1 %) [24], but higher than a report from Wonji Shoa (15.5 %) [20]. In this study, infection with one or more helminths increases as age increases which is in agreement with studies conducted in Ethiopia and Kenya [20, 25].

In the present study, the prevalence of *S. mansoni* infections was (11.2 %). This is in agreement with previous study carried out in Sierra Leone (11.2 %) [26]. In contrary, this finding is lower that studies done Methara (29 %) [23], Wondo Genet (37.2 %) [19], Niger (43.8 %) [15], Cote d’Ivoire (25 %) [27], Kenya (17.6 %) [28] and Uganda (39.3 %) [29]. On the other hand, this finding is higher than studies conducted in Tigray (1 %) [24], Wonji Shoa (8.8 %) [20], Ghana (0 %) [30] and Uganda (7 %) [13]. Difference in water source, time of survey, environmental and socio-economic factors may account for this variation. Of the total 45 PSAC who were positive for *S. mansoni* 32 (71.1 %) and 13 (28.9 %), 0 % had light moderate and heavy infection respectively. However, a report from Niger [15] showed that 17.3, 23.8, 2.7 %,of PSAC were light, moderate and heavily infected respectively. No a statistically significant association was observed between prevalence of *S. mansoni* and age of PSAC in the current study. This is in contrast with a study conducted in Wonji Shoa [20] showing statistically significant association between *S.mansoni* and age of PSAC. Relatively higher prevalence of *S. mansoni* infection in the present study was observed. Therefore including praziquantel treatment in the deworming program as per the WHO guidelines would be essential to decrease the burden of these diseases in this age group [17].

In the current, the overall prevalence of STH infection was found to be 22.9 %. *A. lumbricoides* (19.2 %) was the predominant parasite. This finding was in agreement with a report from Sierra Leone (17.2 %) [26]. However, this is lower than similar reports from Wondo Genet (25.7 %) [19] and Methara (40 %) [23], and higher than a study conducted in Wonji Shoa (4.6 %) [20], Cote d’Ivoire (3.1 %) [27] and Uganda (0.7 %) [13]. The prevalence of *T. trichiura* in the present study was 1.7 %, which is much more lower than studies carried out in Methara (67 %) [23] and Wondo Genet (74.7 %) [19]. The high tolerance of parasites’ eggs and larval stages to the variation of the soil temperature has been described as a key factor for the high transmission and prevalence of these parasites in the area [12].

The majority of PSAC mothers were not heard of intestinal schistosomiasis, 324 (80.8 %) and STH, 235 (58.6 %). Similar to the current study, a study conducted in Malawi reported that the awareness of mother’s about schistosomiasis was very poor, 97 % of the parents had little or no knowledge of the disease [31].

Even though it was not statistically significant in multivariate logistic regression (but significant in bivariate analysis), the habit of washing children in the river increases the prevalence of *S.mansoni*. Another study conducted in Cote d’Ivoire showed that children staying at home with their elders found 2 times more likely to be positive for *S. mansoni* than those who were accompanying their mothers during daily livelihood activities [27]. Both of these findings indicated that PSAC are at higher risk of infection with *S. mansoni.* However, by focusing treatment upon the school-aged population, World Health Assembly (WHA) resolution 54.19 neglects PSAC, thus preventing them from benefiting from the praziquantel treatment given to their older peers, and hence creating a potential health inequity [4].

In the present study, the greater proportion of the mothers of PSAC bring children to the river,(50.9 %) and washed them in the river (47.9 %). This is in line with a study conducted in Niger [15], the majorities of mothers (76.3 %) were accompanied by their children to the canal or the pond and washed them with the contaminated water.

### Limitation

The major limitation of this study is that prevalence and infection intensity of STHs and *S. mansoni* were determined by examination of single stool specimen from each study participants. Thus, we could not access the intra and inter-stool variation of egg output. Furthermore, a single Kato-Katz template was examined for each of the stool specimens that might affect the accuracy of the egg count. Moreover, we could not include all modifiable risk factors in this study.

## Conclusion

The current study has demonstrated that intestinal helmenthiasis and Schistosomiasis were prevalent in varying degree among PSAC in the study area. Maternal habit of not washing hands after toilet, being housewife maternal occupation, the use of protected spring water as a main family source of water and child habit of not wearing shoe were significantly associated with high prevalence of soil-transmitted helminthiasis.. The study also revealed that mothers of PSAC in the study area had limited knowledge on ways of transmission, symptom of the disease and preventive methods of *S. mansoni*. It is, therefore, important to consider including PSAC in ongoing public-health interventions such as the Expanded Programme on Immunization (EPI) and praziquantel mass drug administration (PZQ-MDA) programmes because they are set to suffer from the significant morbidity effects of chronic schistosomiasis. It is important to provide health education for behavior change to avoid contaminating water for the whole village community. It is also advisable to design and implement strategies that enhance access to education and safe water to reduce the burden of soil-transmitted helminthiasis and schistosomiaisis.
